# Health extension programme unit for optimizing access to quality healthcare service in Ethiopia: a case study

**DOI:** 10.1017/S1463423626101030

**Published:** 2026-02-27

**Authors:** Chala Tesfaye, Biruk Bogale, Agumasie Semahegn, Gizachew Tadele Tiruneh, Addis Girma, Rediet Daniel, Kassahun Sime Geleta, Mebrie Belete, Nebreed Fesseha Zemichael, Dessalew Emaway Altaye, Temesgen Ayehu

**Affiliations:** 1 JSI Research and Training Institute, Inc., Boston, USA; 2 AMREF: Amref Health Africa, Kenya

**Keywords:** community health program, Ethiopia, health extension program, primary healthcare

## Abstract

**Background:**

Ethiopia has been working to achieve universal health coverage through optimizing the Health Extension Programme (HEP). The HEP optimization aspires to increase health service access, quality, and equity through different strategies, including establishing HEP units in health centres and primary hospitals. Therefore, understanding the processes of the HEP unit and its implementation experience is crucial for scale-up and sustainability.

**Aim:**

This paper aims to document and share the lessons learned from implementing the HEP unit.

**Method:**

This research collected qualitative data from 14 districts/woredas in 2023. Forty-three in-depth interviews (IDIs) and four focus group discussions (FGDs) were conducted. Audio-recorded data were transcribed verbatim and translated. A thematic analysis approach was used to analyze the data, and direct quotations were used to present the findings.

**Result:**

In the Improve Primary Health Care Service Delivery (IPHCSD) project implementation sites, all 64 health centres, and primary hospitals established HEP units. Setting up the unit improved healthcare provision by promoting collaboration and teamwork, enhancing their skills, coordination, technical support to the catchment health post and increased access to healthcare services through outreach delivery. However, challenges such as a shortage of human resources, dedicated offices for the unit coordinators and team members, inadequate stakeholders’ engagement in the establishment processes, and insufficient tools and supplies were identified.

**Conclusion:**

The HEP unit has improved community-level health services, enhanced health professionals’ skills and teamwork, and technical support to catchment health posts. Strengthening community engagement, advocacy, mentorship, training, and ensuring sufficient staffing, infrastructure, and supplies are essential for the programme’s scale-up and sustainability.

## Background

The Ethiopian government has been investing to ensure universal health coverage by improving the primary health care service delivery through its flagship, the HEP (Ethiopian Ministry of Health, 2020). The HEP is a national community-based health programme designed to improve access to basic health services (Croke, 2020). It was introduced in 2003, and currently, over 40,000 female Health Extension Workers (HEW) who are the primary implementers of the programme have been deployed in more than 18,000 health posts. Each health post provides basic community-based services for a catchment area population of 5000. The HEP is an innovative programme to enhance equitable access to essential health services packages focused on reproductive, maternal, newborn, and child health (RMNCH), disease control, hygiene, environmental health, and health education for communities. (Ethiopian Ministry of Health, 2021). It is a dynamic programme that shifts tasks from health facilities to communities, substantially impacting the community’s health outcomes. (Alemayehu *et al.*, 2023; Yitbarek *et al.*, 2023). Through the programme, Ethiopia has significantly improved maternal and child health, infectious disease prevention and control, hygiene and sanitation practises, knowledge, and health care seeking of the community in rural and hard-to-reach (Gebrehiwot *et al.*, 2015; Assefa *et al.*, 2019; Rieger *et al.*, 2019).

Despite these successes, the programme has faced several challenges including the low performance of the HEWs; poor infrastructure; and rapid demographic and epidemiological disease transition which demands a systemic approach that involves the wider health system, community, and other stakeholders (Sebastian and Lemma, 2010; Yitbarek *et al.*, 2023; Zerfu *et al.*, 2023). Furthermore, care coordination, including planning and service provision with multidisciplinary teams such as nurses, midwives, and health officers, was inadequate (Ethiopian Ministry of Health, 2020). The effectiveness of the HEP is compromised by a weak support system, characterized by inadequate supervision, limited capacity-building opportunities, and insufficient logistical and technical support for HEWs (Banteyerga, 2011). As one of the pillar initiatives, the roadmap envisions restructuring HEP service delivery. This involves upgrading the furthest health posts from the supervising health centres or primary hospitals into comprehensive health posts that can offer advanced community health services. It also suggests merging health posts that are located too close to the health centres and primary hospitals. Furthermore, it recommends setting up HEP units in health centres and primary hospitals to integrate community health programmes for seamless coordination and continuity of health promotion and disease prevention activities within the health facilities and outreach community health services for the catchment communities. The unit is also supposed to provide technical and administrative oversight for health posts under its catchments (Hierink *et al*., 2023). All health centres and primary hospitals establish the HEP unit consisting of 3–5 multi-disciplinary staff. The HEP unit can be established in two different ways: with the HEWs or without them. When health posts are merged, the HEWs become part of the unit’s members. If the health posts are not merged, the health centre or primary hospital will form the HEP unit using the available staff (Ethiopian Ministry of Health, 2020).

Since 2021, the Ministry of Health (MOH) has started implementing the HEP unit initiative as part of the broader HEP service restructuring by designing implementation guidelines and providing training to stakeholders (JSI & Amref IPHCSD Project, 2022). JSI and Amref Health Africa have been providing support to the MOH, regional health bureaus, and woreda (i.e., district) health offices by piloting the HEP unit approach through the ‘IPHCSD’ project. The project tested the HEP unit in 64 health centres and primary hospitals in 14 woredas in pastoralist and agrarian communities since March 2022. Therefore, this paper aims to document and share the lessons learned from the HEP unit implementation, including its success, challenges, and recommendations for future scaling up.

## Methods

### Study design

The study employed a qualitative process evaluation to measure implementation fidelity and document lessons learned from the HEP unit implementation across both agrarian and pastoralist contexts in Ethiopia.

### Study setting

Ethiopia has three tiers/levels of healthcare: primary, secondary, and tertiary care. In the rural context, the primary level of care consists of primary hospitals, health centres, and health posts. The health centres and health posts serve 15,000–25,000 and 3000–5000 people, respectively. Health posts could be either comprehensive or basic and mainly provide various preventive and health promotion services, in addition to treating cases such as malaria, pneumonia, diarrhoea, and other mild illnesses. Health centres provide both preventive and curative services and also serve as referral centres and practical training sites for HEWs. Primary hospitals offer inpatient and ambulatory services to about 100,000 people and also provide emergency surgery (including caesarean sections and blood transfusions). These health facilities form the foundation of primary health care systems and are expected to operate cohesively as a unit, and are called Primary Healthcare Units (PHCUs). The PHCUs are linked with referral and provide capacity building and supervision for health facilities under their catchments. General hospitals are categorized under the second tier of health care and serve on average 1 million people. They are referral centres for primary hospitals and training centres for health officers, nurses, and emergency surgeons. The third tier in the Ethiopian health care system, tertiary health care, consists of a specialized hospital that covers a population of approximately 5 million (World Health Organization, 2017; Assefa *et al.*, 2019; Ethiopian Ministry of Health, 2021). The pastoral settings are known for their mobility and small population size, but inadequate infrastructure makes it difficult to access health services. Compared with the pastoral woredas, the agrarian woredas have better infrastructure and access to health services

### Study participants and sampling strategy

Study participants were purposively selected from four of the project intervention woredas: Seka Chekorsa and South Bench woredas from Oromia and Southwest Ethiopia regions, respectively, to represent the agrarian setting, and two woredas from pastoral: Awash Fentale and Dasench from Afar and South Ethiopia regions, respectively. The study participants were recruited from two primary hospitals, eight health centres, and six health posts from the woredas. The health facilities were selected purposively based on their experience in implementing the HEP unit. Forty-three in-depth interviews (IDIs) and four FGDs were conducted, with 6–12 participants per group for the FGDs.

### Data collection method and tools

Data were collected through IDIs, FGDs, and facility observations in September 2023.

***Facility observation:*** A standardized checklist was used to ensure uniformity across all sites. The checklist covered aspects such as the availability of a dedicated office, equipment, basic profiles, and data management systems.

***IDI:*** A semi-structured interview guide was used to collect in-depth information from participants who were involved in the HEP service restructuring, including categorization of health posts and establishment of HEP units. The participants were selected from woreda health offices, health centres, primary hospitals, and health posts.

***FGDs:*** A discussion guide was used to facilitate FGDs with community members who are beneficiaries of the HEP services and the HEP unit. The FGDs were conducted to explore the perception of the community, experiences, preferences, and suggestions regarding the HEP unit. Local languages (Amharic, Afaan Oromo, and Afar af) were used to conduct the discussions.

Eight interviewers with experience in qualitative data collection and a background in public health participated in the data collection. Data were collected at the participants’ natural setting, where interviews were conducted with healthcare providers, HEP coordinators, and programme managers at their respective offices, and the focus group discussions within the community.

### Trustworthiness

To enhance the trustworthiness of the study findings, a range of techniques were employed. Initially, a semi-structured interview guide was crafted to align with the study’s objectives and thematic areas. Skilled qualitative researchers with substantial experience in the HEP and the study context were selected to lead interviews and FGDs. Sufficient time was allocated for data collection, with interviews typically lasting over an hour and FGDs around two hours, allowing for comprehensive discussions. Daily debriefings were conducted with data collectors to reflect on emerging insights, determine when data saturation was reached, and fine-tune subsequent inquiries. Data credibility was further strengthened through the triangulation of methods (IDI and FGD) and participants. Audio recordings of the sessions were transcribed and meticulously coded using a standardized codebook. To validate the findings, transcripts and preliminary interpretations were reviewed by individuals involved in the study, and differing viewpoints were addressed through collaborative discussion. Finally, programme managers with expertise in qualitative methods and programme implementation reviewed and verified the results to ensure accuracy and rigour.

### Data analysis

The data were analyzed through a deductive and inductive thematic analysis approach with a codebook reflecting the themes and sub-themes. Debriefings on a daily basis with data collectors were conducted to ensure data saturation, completeness, and consistency with the simultaneous incorporation of data from field notes. The tape audio recorded in-depth interviews and FGDs was transcribed verbatim. The transcriptions were translated back to English from the local language for coding and thematic analysis. Iterative reading and subsequent classification were done to classify the data into one of the themes and sub-themes.

### Ethical consideration

This study is part of the larger embedded implementation research, and its protocol was ethically approved by the Ethiopian Public Health Association (EPHA) Institutional Ethical Review Board with Ref #: EPHA/OG/728/23, dated 17 July 2023. The participation in this study was voluntary. Informed verbal consent was obtained from all participants, ensuring they were fully aware of the study’s purpose and their rights. Confidentiality and privacy were upheld throughout the process, including the audio-recorded data and transcripts. The woredas and health facilities were communicated with before the data collection.

## Results

### Characteristics of study participants

A total of 43 study participants (Male = 24 and Female = 19) were interviewed and four FGDs were conducted with the community members (Table 1).

**Table 1.** Characteristics of the study participants, September 2024

Institutions	Number of participants		
	Male	Female	Total
Woreda health office head	3	1	4
Woreda HEP coordinator	3	1	4
Health centre director	8	0	8
HEP unit coordinators	3	6	9
Primary hospital CEO	2	0	2
HEWs at a basic health post	0	6	6
Kebele administrator	3	0	3
Community members (VHLs, WDUs, Religious leaders)	2	5	7
Total	24	19	43

CEO: Chief executive officer; VHLs: Village health leaders; WDUs: Women development unions.

## Identified themes and sub-themes

This study identified several programme implementation successes and challenges that inform the scale-up of the programme throughout the country. The major themes that emerged from the data analysis included 1) HEP establishment processes, 2) the Functionality and performance of the HEP unit, 3) Health workers’ and communities’ perceptions and acceptance, and 5) implementation challenges. (Table 2)

**Table 2.** Major themes and sub-themes emerged from the study, September 2024

Themes	Sub-themes
Implementation processes	Context assessment Training Advocacy meetings with stakeholders
Performance of the HEP unit	Community health service delivery approaches and access Accountability for community health services Linkage among the primary healthcare facilities
Health workers’ and communities’ perceptions and acceptance	Health workers’ perceptions and acceptance Community acceptance and satisfaction
Ongoing implementation challenges	Infrastructure Human resources Community engagement and acceptance

## Implementation processes

The establishment of the HEP units went through assessment, training, and advocacy meetings with stakeholders per the implementation guideline.

**Context assessment:** All health facilities conducted an assessment to categorize the health posts using certain criteria. The criteria for this categorization included the availability and readiness of infrastructure, the distance of the health posts from the supervising health facilities, disease burden in the community, population density, and local government plans to construct a health centre or hospital in the kebele.

Distance was a key criterion to merge health posts into catchment health centres or primary hospitals. Health posts located within the same kebele or one hour walking distance from the supervising health facility were merged with the health facility, and the HEWs became a part of the unit. Health posts situated far from the supervising health facilities and with high catchment populations were prioritized for comprehensive types of health posts, while the remaining health posts within a reasonable distance from the supervising health centres or primary hospitals were classified as basic and expected to provide quality community health services.

This categorization system helped the HEP unit to prioritize and plan for tailored support to catchment health posts, avoid duplication of services in the community, and establish and map HEP unit staff.

**Training:** Training was provided to the staff to have a shared understanding of the HEP unit establishment processes and roles in all project woredas. The key informant reported that the training helped them understand and establish the HEP unit.

*‘… my colleagues and I were the first to take the training [regarding HEP roadmap implementation] …then we oriented the health center staff, provided necessary manuals to the staff… After that, we established the HEP unit …’* **Woreda Health Office Head**.

Similarly, the training covered the roles and responsibilities of HEP unit staff, an introduction to tools for measuring the unit’s functionality, and the criteria required for its establishment. This training has been instrumental in highlighting the crucial role of HEWs and other staff in implementing the HEP units, especially where there is a merged health post as per the criteria.

**Advocacy meeting with stakeholders:** Advocacy meetings were held with various stakeholders, such as local administrators, health centres and primary hospitals, health management members, HEWs, religious, clan leaders, and community representatives. These meetings aimed to discuss the major changes and the importance of establishing HEP units to improve community health services. Additionally, public conferences were organized in some areas to gather community input on restructuring health programmes. The consultative meeting helped to share the plan, roles, and responsibilities of different stakeholders and facilitate the establishment of the HEP units.

### Performance of the HEP units

The HEP units were equipped to provide comprehensive and effective health services to the community and robust technical support for health posts, as evidenced by the qualitative inquiry. The programme implementers witnessed that the HEP unit strengthened community health programmes, governance and accountability, links between primary-level healthcare facilities, and improved the performance and efficiency of the primary healthcare system.

**Improved community health service delivery approaches and access:** The establishment of the HEP unit has enhanced team-based service delivery to the community, which is important to the provision of quality services. For instance, the HEP units have initiated outreach services, where midwives and nurses have actively participated in delivering essential healthcare services, such as conducting pregnant women’s conferences, immunization, nutritional screening, and family planning. The healthcare workers have reported that the HEP unit has significantly improved the delivery of disease prevention and health promotion packages. The unit has also promoted team-based service provision at the facility level, even though there are gaps in the implementation.

*’The HEWs are assigned to perform their activities at static and outreach models. They work two days in the health center and three days in the community. At the health center, they provide child vaccination services, maternal follow-up visits, and family planning services’.* **Health Centre Director**

**Improved accountability for community health programme**: Each healthcare facility has designated a coordinator for the unit to oversee the implementation of their assigned roles, thus providing effective coordination for the community health programme. According to the key informant from the Health Centre, the HEP unit has improved coordination and accountability.

*‘Previously, all of these activities [community health program activities] were managed under the health center director and sometimes by the health extension coordinator. As the roles of the director and coordinator were mixed, there was a time when the community health program was neglected. Now, since the establishment of the HEP unit, the coordinator takes the responsibility of coordinating the community health activities’.* **Health Centre Director**

**Strengthened linkages between primary-level healthcare facilities:** The implementation of the HEP facilitated better communication and collaboration between healthcare workers, support systems, and referral networks. A two-way referral and feedback system between the village health leaders (VHLs) to the health posts, the health posts to the health centre, and the primary hospitals was established. They reported that this unit was also serving as a common platform for facilities to share their experiences.

*‘Previously, the linkage of the health post and health center was weak, but currently, there is a big improvement in technical support, both in terms of frequency and content of the supervision, that improved our skills. The previous one focused on performance evaluation, but nowadays they support us in all aspects. For example, I am alone working in this kebele; they are supporting me to improve my performance and my skills as well’.* – **HEW**

**Improved quality of service delivery:** The establishment of the HEP units has significantly improved the capacity of healthcare providers to provide essential health services at the health centres. Furthermore, the performance of HEWs on community-based outreach and home visit services has been improved due to their enhanced skills, motivation, and better support by the unit. The implementation of a team-based service delivery approach has led to a significant improvement in the quality of healthcare service provision since healthcare workers collaborate, share their skills, and provide a wide range of services in an integrated approach.

**Health workers’ and communities’ perceptions and acceptance**: The establishment of HEP units was perceived positively among health workers and community members.

**Improved technical support to the HEWs:** The programme significantly enhanced HEWs’ skills and fostered better collaboration with other health workers working at the health centre. The unit members provide technical support in undertaking community mobilization, such as training of VHLs and Women Development Unions (WDUs).

*‘… since they joined the health center, the HEWs’ skills have improved. They are advancing their skills by working together in different areas such as family planning, EPI, and ANC’.* **-Health Centre Director**

*‘… the HEP unit supported me in recruiting and deploying VHLs in four gots [sub-villages]. …I was not engaged at work before, but now I have engaged well with a new moral and energy’.* **HEW**

**Community acceptance and satisfaction:** The community accepted the establishment of the HEP unit and the provision of community health services through the team-based approach, especially in areas where the health posts and health centers were closer to each other. Before the establishment of the HEP unit, the health centres’ proximity to the health posts made it the preferred choice for community members, resulting in underutilized health posts.

*‘… the health posts are adjacent to the health center. From the beginning, the community chose the health center because it is close to the health center’.* – **Health Centre Director.**

One community member expressed that, before the establishment of the HEP unit, there was a lack of consistent follow-up on community health issues. However, with the unit in place, they observed a meaningful change – health workers and local leaders now collaborate more actively, holding regular discussions to address community concerns. This collaborative approach has made residents feel acknowledged and respected, fostering a stronger sense of trust in the health system.

*‘…………. Now we see the health workers and leaders coming together, discussing our issues, and solving them. We feel heard and respected’.* – **community member**

### Implementation challenges

The implementation of the HEP units encountered various challenges. These challenges included shortages of human resources, budget, facility infrastructure, supplies, equipment, and suboptimal commitment and guidance for its implementation.

### Poor infrastructure

The lack of adequate infrastructure, such as a dedicated office, was the most frequently raised challenge at the health centre. Resource constraints also limited the functionality of the HEP, as there were difficulties in equipping and furnishing it.

*‘The unit has challenges. It has a shortage of infrastructure, including a dedicated office for the unit members. This creates gaps for members to meet and review activities together’.* – **Health Centre Director**

Furthermore, shortages of stationery, file storage shelves, and medical equipment for the unit and health posts were the major challenges in the implementation of the unit.

### Shortage of human resources

The shortage of health professionals and high staff turnover exacerbated the problem. As a result, HEWs often had to work alone during outreach services. There was ambiguity in the implementation of the HEP unit guidelines due to inadequate training, which made teamwork difficult among some staff. Even though there is some support, more effort is needed to get the required commitment from health leadership, who often have competing priorities and demanding schedules.

### Low community engagement

Engaging all community members to actively participate in the consultation processes was not possible in some areas. In a few areas, some community members prefer to have health posts, despite their proximity to the health centre or hospital. Some respondents mentioned that during the initial phase, a drop in community health services such as vaccination and family planning has been observed as a result of merging the nearby health posts and inadequate orientation to the community members.

Participants in pastoral settings mentioned that the community was hesitant regarding merging health posts in the early phase of the process. The community mentioned that the health posts are relatively nearer to them and have less waiting time.

*‘…during the initial phase, there were objections from the community members who were served from the health post, but advocacy work was done with the community, health extension workers, and kebele administrators on the importance of merging health posts…’* – **HEP Unit Coordinator**

Similarly, another key informant mentioned that,

*‘…in the initial phase, it was somewhat frustrating for the community, some of them even called me over the phone … complained why it was closed. I told them as it is for better service …and when they saw the service being given here in the health center, they are now happy’.* **HEW**

## Discussion

This study identified that the establishment of the HEP unit through a participatory and contextual manner has been accepted by the community and improved skills and motivation of the HEWs, and the delivery of health services.

Stakeholders’ engagement and advocacy on the establishment of the HEP unit were critical to ensure the acceptability by the communities and health workers. This study highlighted that engagement of local administration, Health centres, hospital management members, HEWs, religious leaders, clan leaders, and community representatives facilitated the implementation of the programme. Evidence showed that engaging communities proactively in the planning, design, delivery, and evaluation of primary health care services can lead to improved community health (Neuwelt, 2012; Erku *et al.*, 2023). However, there are incidents of community resistance at the initial phase of implementation of the HEP unit establishment in pastoralist areas, which is linked to the fear of extra distance to access health services. Addressing such legitimate concerns from the community through discussion and consensus building, and implementation of the merging criteria is crucial for the success of the programme implementation and sustainability. Moreover, successful stakeholders’ engagement in the aim, merging, and role could foster representation and transparency on the initiatives for the sustainability of the programme (Masefield *et al.*, 2021; Petkovic *et al.*, 2023).

The HEP unit has improved health service delivery. Additionally, the interdisciplinary team of the unit gave HEWs the abilities and know-how needed to offer health education and basic medical treatments. This strategy might make health promotion and education an essential component of the delivery of health services and further improve the efficient integration of health education approaches into the stages of primary health care service planning, delivery, and monitoring. As evidenced by previous studies, such an approach could bridge the gap in the transfer of educational and planning skills to community health workers and the community at large at the health facilities (Wendimagegn and Bezuidenhout, 2019). Additionally, community-based health services were instrumental in increasing accessibility and improving health outcomes in rural and hard-to-reach areas, where access and efficiency were most needed (Mangham-Jefferies *et al.*, 2014).

The establishment of the HEP unit and provision of community health services through a multidisciplinary team composed of nurses, midwives, health officers, and HEW, with active community engagements through platforms such as the VHLs and the WDUs, has improved health promotion and disease prevention activities and provided better technical support to catchment health posts. In addition, the service provision has improved due to the multidisciplinary team composition. This is in line with the previous evidence that an effective multidisciplinary team enhanced the demand creation, engagement, and community ownership to take responsibility for their health (Dussault *et al*., 2018; Saint-Pierre *et al.*, 2018). Another study conducted by Leach et al. (2017) found that interprofessional collaboration was associated with improvements in patient outcomes, service delivery process, and patient satisfaction (Leach *et al.*, 2017).

Further, the programme created a platform for cross-learning and experience sharing within the team that enhances their skills, motivation, and capacity. Health extension workers were able to acquire new knowledge, fill gaps in their existing knowledge, and learn new skills from other healthcare providers. Previous studies have shown the effectiveness of integrating community health workers (CHWs) into multidisciplinary teams to reduce health disparities among vulnerable and underserved populations (Islam *et al.*, 2015; Knowles *et al.*, 2023; Van Iseghem *et al.*, 2023). Our finding indicates that integrating HEWs with a multidisciplinary team of midwives, Nurses, and Health Officers fosters teamwork, knowledge sharing, and capacity in providing healthcare services for the community.

One of the goals of the HEP unit is to improve the performance and efficiency of the primary healthcare system. (Hierink *et al.*, 2023). Our study confirms that after the establishment of the HEP unit, human resource efficiency improved and optimized the community-based service delivery. Previous evidence showed that supportive supervision and mentorship have a significant effect on improving the primary healthcare service delivery (Islam *et al.*, 2015). Similarly, the HEP unit demonstrated improved service delivery, where the integration of the HEWs with allied healthcare providers enhanced the efficiency of the outreach programmes and community-level service delivery (Quinlan and Robertson, 2010; Leach *et al.*, 2017; Saint-Pierre *et al.*, 2018).

Despite the promising outcomes of HEP units’ establishment in the health centres and primary hospitals, there were implementation challenges to be addressed. Lack of adequate infrastructure, including dedicated rooms, supplies, and equipment, was the most frequently raised challenge, which limited the full functionality of the HEP unit. Evidence in Ethiopia shows a significant number of health centers and health posts lack the basic infrastructure to provide quality curative and preventive health care services (Assefa *et al.*, 2019). It is imperative to improve the health facility’s basic infrastructure through advocacy for increased funding by the relevant stakeholders.

Shortage of adequate human resources, turnover of staff, and inadequate guidance on the programme were also among the major challenges. It is evident in previous studies that the Ethiopian healthcare system falls short of the WHO’s benchmarks for population density to provide quality health services (Hierink *et al.*, 2023). These emphasize the need for additional investment to recruit and retain health workers to improve the staffing of the HEP units and the entire primary health care system. Finally, support from the leadership at all levels is very critical to allocate resources, which remains to be improved through ensuring accountability.

### Strengths and limitations of the study

To the best knowledge of the authors, this is the first study to explore the implementation of the HEP unit establishment in both agrarian and pastoral settings of Ethiopia, offering valuable insights for further improvement. Further, the study included diverse views of stakeholders and experts working at the primary healthcare level. However, since the study was conducted at the early establishment of the HEP unit, it might not have assessed the full spectrum of the programme. Moreover, individual circumstances may have an impact on qualitative findings, and subjectivity may be introduced into the analysis.

## Conclusion

The establishment of the HEP unit demonstrated the value of a participatory and integrated approach to improving primary healthcare. By engaging stakeholders through assessments and training, the initiative fostered multidisciplinary collaboration, enhanced referral systems, and improved service accessibility and quality delivery. However, key challenges –– such as limited human resources, infrastructure gaps, inadequate operational guidelines, and weak stakeholder engagement – hindered full implementation and impact. These barriers point to systemic issues that require targeted policy attention and strategic planning.

To ensure sustainability and scale-up, it is essential to strengthen advocacy and community engagement, build the capacity of health workers through structured mentorship and training, and invest in recruitment and retention strategies. Policymakers should prioritize resource allocation for infrastructure, develop clear operational guidelines, and establish robust coordination mechanisms among sectors. Promoting data-driven decision-making and accountability at all levels of the health system will be crucial for improving performance and ensuring that the HEP unit model contributes meaningfully to universal health coverage goals.

## Data Availability

Data is accessible upon request and may be shared, provided the intended purpose is clearly outlined. For inquiries, please contact the corresponding author at chalatesfaye@yahoo.com.
